# 

**Published:** 1874-08

**Authors:** 


### EDITORIAL:

Obituary .................................................. 359
Resolutions of Respect adopted by the Dentists of Nashville to the late Dr. Hamlin ....................... 360

### THE DENTAL REGISTER,

### A Monthly Magazine devoted entirely to the Interest of

### THE DENTAL PROFESSION.

**Terms Reduced to $2 50 per Year. Single Numbers 25cts.**

**EDITED BY PROF. J. TAFT,**

**And Published by SPENCER, CROCKER & CO., Ohio Dental Depot.**

The volume commences in January and closes in December.

The REGISTER being issued monthly is always fresh, therefore indispensable to the practitioner who desires to keep posted up with the new inventions and improvements which are daily developed. Neither expense nor effort will be spared to give value and variety to its contents. Articles will be illustrated with well executed engravings whenever they will add to the interest or assist in explanation.

Its extensive circulation and frequency of issue make it one of the BEST DENTAL ADVERTISING MEDIUMS in the United States.

All subscriptions must begin with January or July.

**RATES OF ADVERTISING.**

| | 1 Mo. | 3 Mo. | 6 Mo. | 1 Year. | | 1 Year. | |
|---|---|---|---|---|---|---|---|
| 1 page.... | $12 00 | $25 00 | $40 00 | $75 00 | Inserted pages must | 1st page. | $50 00 |
| ½ page.... | 8 00 | 15 00 | 25 00 | 40 00 | be plain paper and | 2d page. | 37 50 |
| ¼ page.... | 5 00 | 10 00 | 15 00 | 25 00 | black ink. | 3d page. | 33 33 |
| | | | | | | 4th page. | 25 00 |

Local or special notices, five lines or less, will be inserted for $3 each insertion: over five lines, 50 cts. per line

All advertising bills payable in advance, or at furthest, after the issue of the first number containing the advertisement. All transient advertisements to be paid for in advance.

☞ CONTRIBUTIONS SOLICITED.

Exclusively Editorial Communications should be addressed to

**DR. J. TAFT, EDITOR.**

The latest number of the REGISTER may be ordered with or without other goods at any time, and all letters should be addressed to

**SPENCER, CROCKER & CO., OHIO DENTAL DEPOT,**
Cincinnati, Ohio.

